# Identification of Buried Objects in GPR Using Amplitude Modulated Signals Extracted from Multiresolution Monogenic Signal Analysis

**DOI:** 10.3390/s151229801

**Published:** 2015-12-04

**Authors:** Lihong Qiao, Yao Qin, Xiaozhen Ren, Qifu Wang

**Affiliations:** 1 College of Information Science and Engineering, Henan University of Technology, Zhengzhou 450001, China; lhqiao@haut.edu.cn (L.Q.); rxz235@163.com (X.Z.); 2 Key Laboratory of Grain Information Processing and Control, Henan University of Technology, Ministry of Education, Zhengzhou 450001, China; 3 Henan Academy of Science, Applied Physics Institute Co., Ltd, Zhengzhou 450001, China; ewangqifu@hotmail.com

**Keywords:** reflection hyperbola, GPR, multiresolution monogenic signal analysis, amplitude component, Hough transform

## Abstract

It is necessary to detect the target reflections in ground penetrating radar (GPR) images, so that surface metal targets can be identified successfully. In order to accurately locate buried metal objects, a novel method called the Multiresolution Monogenic Signal Analysis (MMSA) system is applied in ground penetrating radar (GPR) images. This process includes four steps. First the image is decomposed by the MMSA to extract the amplitude component of the B-scan image. The amplitude component enhances the target reflection and suppresses the direct wave and reflective wave to a large extent. Then we use the region of interest extraction method to locate the genuine target reflections from spurious reflections by calculating the normalized variance of the amplitude component. To find the apexes of the targets, a Hough transform is used in the restricted area. Finally, we estimate the horizontal and vertical position of the target. In terms of buried object detection, the proposed system exhibits promising performance, as shown in the experimental results.

## 1. Introduction

As a geophysical method, ground penetrating radar (GPR) is used in the detection of the targets in near the ground surface areas for locating underground objects such as land mines and pipes. We focus on the metal point detection in this paper. All hyperbolas have to be located and each single radargram has to be analyzed, for the purpose of extracting the exact position of the targets.

There are some researches on buried object detection. To identify hyperbolas, Delbo *et al.* [1] propose a procedure based on wavelets to reduce noise and then a fuzzy cluster approach to detect objects. Artificial neural networks are used in [2]. A pattern recognition process is used to locate underground objects using a pattern recognition process [3,4]. Real-time interpretation of GPR images is proposed in [5].

A model called Multiresolution Monogenic Signal Analysis (MMSA) has been developed by Unser [6]. Multiple components such as amplitude components and phase are obtained using monogenic signal analysis, which has a proper multiresolution presentation. The wavelet-based amplitude components suppress noise and enhance targets in GPR images. Dong *et al.* [7] propose synthetic aperture radar (SAR) target classification method based on Riemannian geometry. Combined with a covariance matrix, the monogenic components are used in SAR image classification. Besides, sparse representation of the monogenic signal of SAR images is proposed in [8].

This paper aims to present a novel method for solving the object detection issues. The method includes preprocessing of the data using an MMSA to extract the amplitude component. After the extraction, background clutter is removed to a large extent while keeping the target signal, which aims to get rid of the false targets and locate the targets accurately. To limit the scope of reflection hyperbolas to certain areas, we use the ROI extraction method in the GPR data, which decreases the computational complexity of hyperbola fitting. Then, the Hough transform is used for hyperbola fitting. Finally, we estimate the horizontal and vertical position of the target. Experiments on GPR images come from the finite-difference time-domain (FDTD) method and realistic situations.

The paper is organized as follows: the reflection model of a buried object is introduced in Section 2. The detection algorithm based on MMSA, extraction of ROI and Hough transform application is introduced in Section 3. The experimental results are illustrated in Section 4. Finally, conclusions are presented in Section 5.

## 2. Reflection Model of a Buried Object

**Figure 1:**
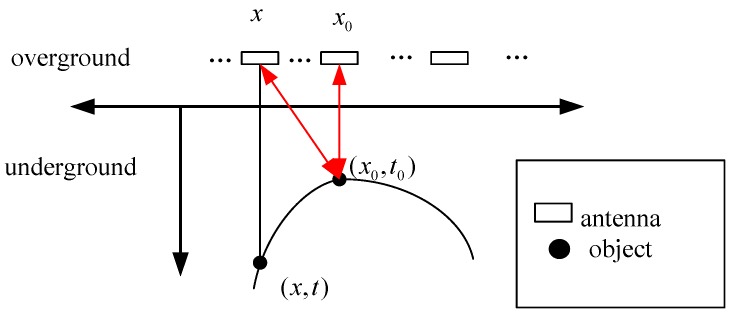
A simple reflection model for GPR. The antenna position x and the echo time delay t approximately satisfy the hyperbolic Equation (1).

A radar system contains a transmitting and receiving antenna pair. First, we review the reflection model of a buried metal point target. When the ground-coupled antenna scans an object linearly, the antenna position x and corresponding echo time delay t approximately satisfy a three-parameter hyperbola equation:

(1) $$\frac{t^{2}}{t_{0}^{2}}-\frac{4{\left(x-x_{0}\right)}^{2}}{v^{2}t_{0}^{2}}=1$$

where v is the velocity of wave propagation underground, assumed to be constant in a small region, $x_{0}$ is the horizontal position of the object, and $t_{0}$ is the time delay for the echo. The apex $\left(x_{0},t_{0}\right)$ indicates the object position of the vertex. The velocity v and the position $\left(x_{0},t_{0}\right)$ of the vertex determine the shape of the reflection hyperbola. A simple reflection model for GPR is shown in Figure 1.

## 3. Detection Algorithm

The object detection method consists of four steps:

1. Extract the amplitude components using the MMSA method;
2. Extract the Region of Interest to narrow down the region to certain areas;
3. Locate hyperbolic patterns via a Hough transformation;
4. Estimate the horizontal and vertical position of the target.

**Figure 2:**
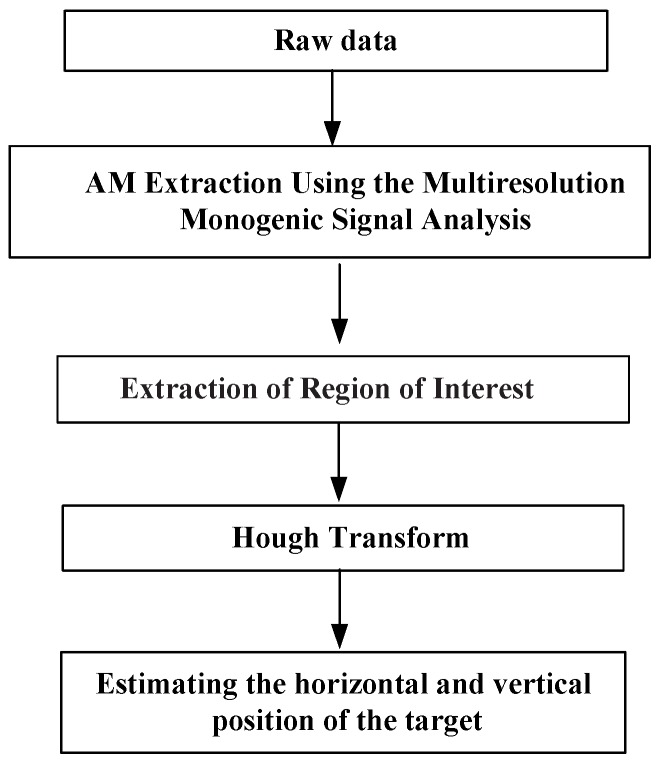
The flowchart of the proposed strategy.

The flowchart is shown in Figure 2. 

In detail, the GPR image is first decomposed by the monogenic wavelet transform to extract the amplitude component. The amplitude component enhances the target reflection and suppresses the direct wave and reflective wave to a large extent. The phase information decomposed by the monogenic wavelet transform contains too much detailed texture information of all waves and it is hard to locate the target. The method can not only intensify the target, but also eliminate the undesired presence of the ground surface echo. Then we use the ROI extraction method to restrict the region to a small range. The extraction is based on the normalized variance bigger than a fixed number. The computational cost of the Hough transform is significantly reduced. Then the Hough transformation is used to detect hyperbolic patterns.

### 3.1. Preprocessing Based on the Multiresolution Monogenic Signal Analysis Structure

The MMSA method uses the Riesz-Laplace wavelet transforms. Three wavelet components—amplitude components, phase components and instantaneous frequency components—are extracted. These parameters have a very clear definition in radio frequency theory and they can be extended to two dimensional signals. Using the amplitude component, background clutter is removed to large extent while keeping the target signal.

In this section we first review the monogenic signal. Then, we introduce the Riesz-Laplace wavelet transforms to implement the MMSA method and get the modulation characteristics, which include amplitude, phase and frequency.

Three-components of monogenic signal f(χ) are defined in [9] as:

(2) $$\begin{array}{l} f_{m}(\chi )\\ =(f(\chi ),Re(ℜf(\chi )),Im(ℜ(f(\chi ))))\\ =(f,f_{1},f_{2}) \end{array}$$

where f(χ) is a two dimensional signal, χ=(x,y), and ℜ is the Riesz transform of f(χ).

Amplitude component is defined by:

(3) $$A(x)=\left\|f_{m}(x)\right\|=\sqrt{f^{2}+f_{1}^{2}+f_{2}^{2}},$$

The orientation θ and phase ξ are given by:

(4) $$f=A\cos\xi ,f_{1}=A\sin\xi \cos\theta ,f_{2}=A\sin\xi \sin\theta$$

In particular, the amplitude components and phase components are recovered in the direction $u=(\cos\theta ,\sin\theta )=(f_{1}/r,f_{2}/r)$ , which can be extended to other directions.

Then we show how to realize a monogenic representation of wavelet coefficient. On each bandpass-filtered signal:

(5) $$(f*\psi_{i})\left(x\right)$$

where $\psi_{i}\left(x\right)={\left|\det(D)\right|}^{i/2}\psi \left(D^{i}x\right)$is the normalized and spine wavelet. 

Due to the symmetry characteristics, the coefficients of the signal are defined as:

(6) $$\omega_{i}[k]=\langle f,\psi_{i,k}\rangle \text{=}{\left(\psi_{i}*f\right)(x)|}_{x=D^{-(i+1)}k}$$

(7) $$r_{1,i}[k]+jr_{2,i}[k]=\langle f,ℜ*\psi_{i,k}\rangle \text{=}ℜ{\langle f*\psi_{i,k}\rangle |}_{x=D^{-(i+1)}k}$$

where Riesz coefficient’s real component is $r_{1,i}[k]$ and the imaginary component is $r_{2,i}[k]$. k is the space index and i is the scale index.

Based on previous knowledge, the MMSA is applied to extract the amplitude components in the wavelet-domain. The tensor method is used to estimate the local orientation. The corresponding directional Hilbert component is given by:

(8) $$q_{i}[k]=r_{1,i}[k]\cos\theta +r_{2,i}[k]\sin\theta$$

Then the analytic representation is:

(9) $$\omega_{i}[k]+q_{i}[k]j=A_{i}[k]\cdot e^{j\xi_{i}[k]}$$

Then the modulation components are defined by:

(10) $$A_{i}[k]=\sqrt{\omega_{i}{\left[k\right]}^{2}+q_{i}{\left[k\right]}^{2}}$$

(11) $$\xi_{i}[k]=arctan(\frac{q_{i}[k]}{\omega_{i}[k]})$$

We use MMSA to define the amplitude component. The frequency component contains too much noise of the reflective wave and it is not so convenient to use this component. In monogenic representation, the local amplitude represents the local energetic information, whereas the phase depicts the local structural information. Thus, the monogenic signal fulfills the invariant property with respect to energy and orientation.

**Figure 3:**
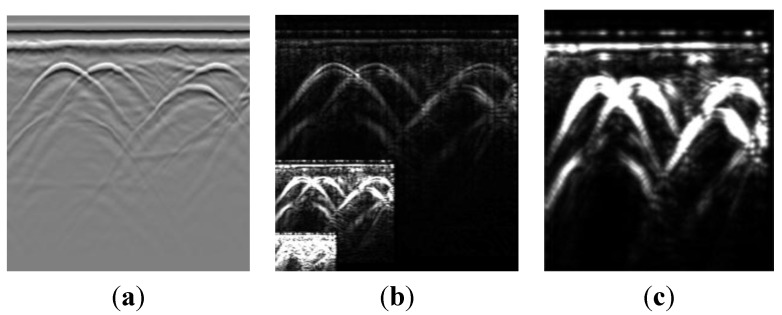
Example of amplitude components extracted by the MMSA method. (**a**) Input image; (**b**) Wavelet-domain amplitude estimation. Three amplitude components under three level monogenic wavelet analysis. The amplitude components eliminate the direct and surface-wave noise; (**c**) The enlarged second amplitude component.

Figure 3 illustrates the ability of the MMSA method to extract the amplitude component. With a fixed Gaussian window, we use a tensor-based method to estimate the orientation. Figure 3a is a GPR experiment data profile. It contains recorded point data with a mean frequency of 300 MHz and a time window of 100 ns. The horizontal and vertical coordinates are the trace and sample points, respectively. The number of traces is 540 and the number of sampling points for each trace is 512. Figure 3b illustrates the three amplitude component image extraction result under three level monogenic signal analysis. Three amplitude components are depicted in one picture. As compared with Figure 3a, the ground surface has obvious direct waves, and the first amplitude component suppresses the direct wave and reflective wave to a large extent. This representation not only enhances the target reflection, but also eliminates the redundant echo. Figure 3c shows the enlarged second amplitude-component. The amplitude component makes the target signals obvious.

### 3.2. Extraction the Region of Interest

Cheng proposed the Region of Interest (ROI) extraction method [10]. Based on the analysis of GPR A-Scan signal’s statistical features, a windowed statistical method is used to extract the ROI from a large amount of data. 

**Figure 4:**
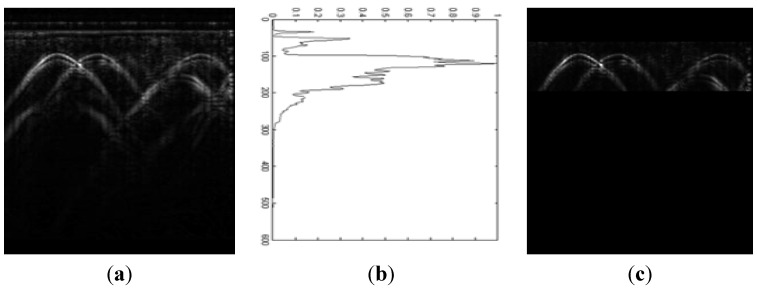
Example of the extraction of Region of Interest. (**a**) The first amplitude modulation component extracted by using the MMSA; (**b**) The normalized variance of amplitude component; (**c**) The extraction of ROI of the amplitude based on the normalized variance with a lower threshold.

In detail, the variance of the B-scan data is calculated. Then the normalized variance of the data is calculated. We note that the variance is significantly larger in the target area. The ROI is extracted based on the normalized variance being bigger than a fixed number. The first amplitude component extracted by the MMSA is shown in Figure 4a. Then the normalized variance of amplitude component is illustrated in Figure 4b. The extraction of ROI of the amplitude is based on the normalized variance with a lower threshold. The extraction result is shown in Figure 4c. This method gets rid of the false targets and locates the targets accurately and it is especially used for the targets at almost the same depth. 

### 3.3. Hough Transform

We briefly introduce the Hough transform as follows. We rearrange Equation (1):

(12) $$t_{0}=\sqrt{t^{2}-\frac{4}{v^{2}}{\left(x-x_{0}\right)}^{2}}$$

where(x,t) represents the reflection hyperbola’s coordinates and $x_{0}$, $t_{0}$, and v are the parameters. We first limit the image area by the ROI extraction algorithm, so the computational time decreases dramatically. The Hough transform used in our paper was shown in [11].

**Figure 5:**
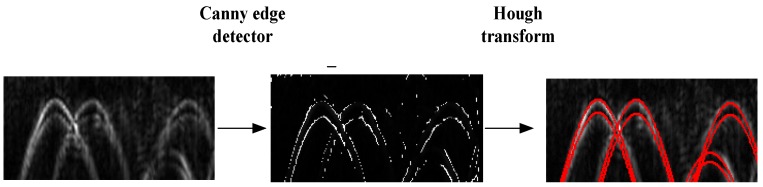
The result of the Hough transform. The image is in the ROI region (on the left). Then the canny edge detects result is shown. Then the Hough transform result. The hyperbola is drawn in red color.

In our method, ROI limits the area before the Hough transform. In order to reduce noise and artifacts, the image in marked regions is smoothed with Gaussian filters. Then by a canny edge detector, this image is converted into a binary image. Then using the Hough transform, the position of the target is determined. In detail we define the number of accumulator-cells for $x_{0}$ and $t_{0}$ using the edge pixels. The propagation velocity is an important parameter to obtain the best fitting. It varies for different soil characteristics especially for large range of areas. The velocity v in the Hough transform varies in a given interval assuming the range is not so big, which can be set by the user. Then we transform every edge pixel (x,t) and every discrete value of v into the Hough plane with Equation (1). Finally, we draw the location result in the original image (see Figure 5). Firstly, using the Gabor filter, the image in the extracted region is smoothed. By a canny detector, the image becomes a binary image (shown in the middle of Figure 5). Then we use Hough transform in the ROI region. The calculated hyperbolas are drawn in red color.

## 4. Results

We use GPR numerical simulation images and experimental images to assess the target detection. The electromagnetic simulator “GprMax” is used to generate simulation images using the finite difference time domain (FDTD) method. RIS radar is applied to collect the experimental data. 

### 4.1. Simulation Results

**Figure 6:**
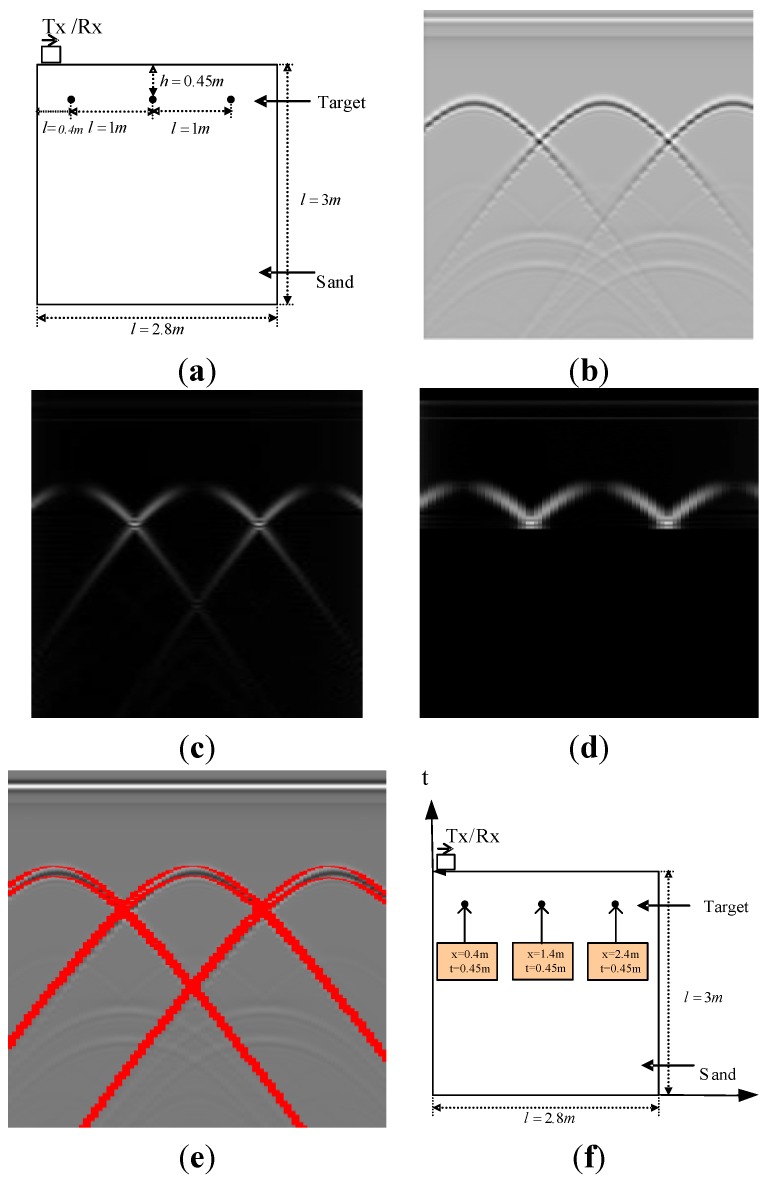
Example of MMSA detection result. (**a**) The location in reality; (**b**) GPR image; (**c**) Extraction of the amplitude component using MMSA; (**d**) Extraction the ROI of the amplitude component; (**e**) The hyperbola result of the Hough transform; (**f**) The position estimation result of the detection method.

The whole simulation process is shown in Figure 6. Figure 6a illustrates a scenario of metal blocks in three distinct steps. This is a simulated image. The width and depth are 2.8 m and 3 m, respectively. The background medium is dry sand and three perfect conductor metal cylinders with 0.01 m diameter are buried at the same depth of 0.45 m. The interval of the three metal targets is 1 m. In the simulation model, the grid in the x and y direction is 0.01 m and detection step is 0.02 m in the x direction. All the boundaries are Perfectly Matched Layers (PMLs). The parameter of the current definition is a Hertzian dipole with unit amplitude and center frequency of a Ricker wavelet of 600 MHz, and the time window for each trace is 25 ns. There are 141 traces in total and 1060 samples. The interval between samples is 2.3587 × 10^−11^ s. The actual electromagnetic wave velocity is 14.8 cm/ns. The related GPR image is shown in Figure 6b which is generated by the “GprMax” simulator. The extraction result of the first amplitude component of the MMSA step is shown in Figure 6c, where the reflection signals and the noise can be suppressed and the target reflection can be enhanced after the extraction of the amplitude component. Then the ROI extraction method is used to get rid of the false targets and locate the targets accurately. The result is illustrated in Figure 6d. The results allow one to the find the three patterns correctly as can be seen in Figure 6e.

**Figure 7:**
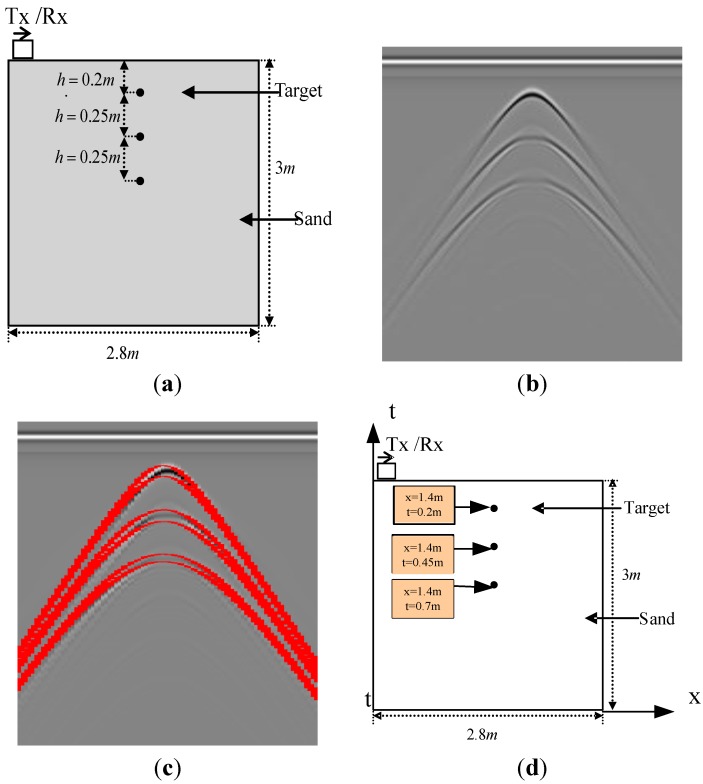
One more example of using MMSA. (**a**) Ground truth of the image; (**b**) GPR image; (**c**) The hyperbola result of the Hough transform; (**d**) The position estimation result of the detection method.

Another result is presented in Figure 7. Figure 7 is an image simulated with the FDTD method. The width and depth are 2.8 m and 3 m, respectively. There are three perfect metal point targets with the same x coordinates. The depth of the top one is 0.2 m below the ground surface and the interval of the other two targets is 0.25 m. The antenna receives a trace for each 2 cm. There are 141 traces in total which contain 1060 samples. The interval between samples is 2.3587 × 10^−11^ s. The actual electromagnetic wave velocity is 14.5 cm/ns. Each detection result is processed by the Hough transform. This method detects the three patterns correctly.

### 4.2. Experiment Results

**Figure 8:**
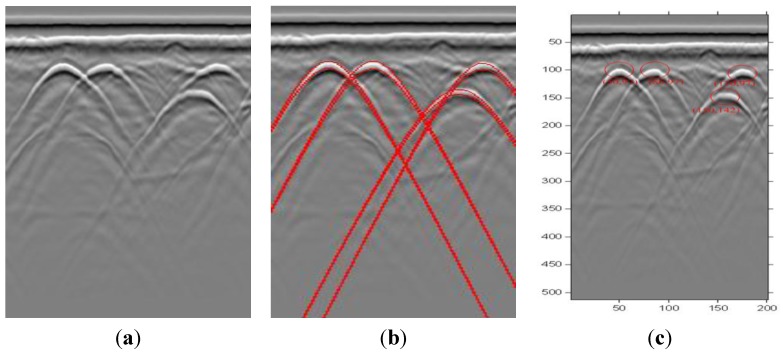
Example of test metal point targets data. (**a**) GPR image; (**b**) Processed result with detected patterns. The detected hyperbola is the red color line; (**c**) The position estimation results of the real point data.

Then we get GPR images in realistic situations. The first example is shown in Figure 8a, which is data from a real GPR experiment. Four metal spheres of 0.02 m diameter were buried under dry sand. These spheres were located side by side. The figure records the detection data with mean frequency of 200 MHz and time window of 80 ns. Finally, the detection result is attached to the picture (see Figure 8b). Further, $x_{0}$ and v are estimated by fitting the extracted hyperbola. After the Hough transform, we get that the velocity of the data is v=1.38 × 10^8^ m/s, which is tested with the position of the targets in the real situation. As illustrated in Figure 8c, there are four targets in the ground. According to the extracted hyperbolic vertex coordinates, the antenna position of the target is shown in Figure 8c. For example, the vertex coordinate of the left target is $x_{1}=50$, so the antenna position of the target is 3 m, which is the same as the position of the target in reality. Besides, the vertex coordinates axis of the hyperbola represents the depth of the target. The vertex coordinate of the left target is $t_{1}=97$.

**Figure 9:**
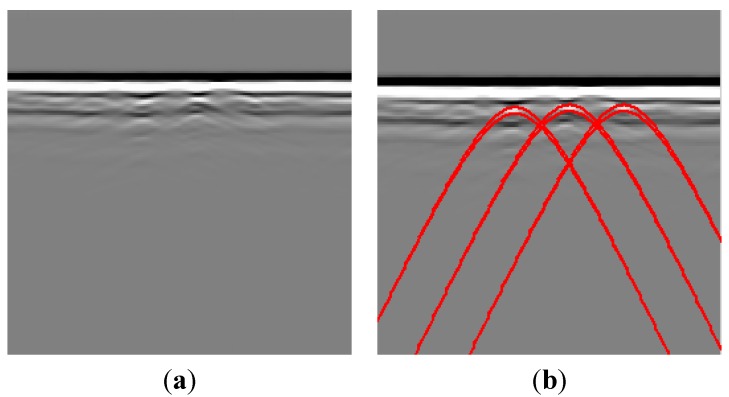
Example of landmine detection results. (**a**) GPR image; (**b**) Processed image with detected patterns.

A second example provided by the MMSA method is shown in Figure 9. Three similar landmines with a plastic case were buried under dry sand. The mines are Type-72A. The diameter of the landmine is 7.7 cm and the height is 3.7 cm. Figure 9a shows the final detection result with the proposed MMSA method. Note that the proposed detection scheme provides a high detection rate even though the target is not so obvious. Figure 9a is a real GPR data profile. It recorded the point data with a mean frequency of 200 MHz and time window of 50 ns. The detection result is shown in Figure 9b, which illustrates that all patterns are identified with high precision.

**Figure 10:**
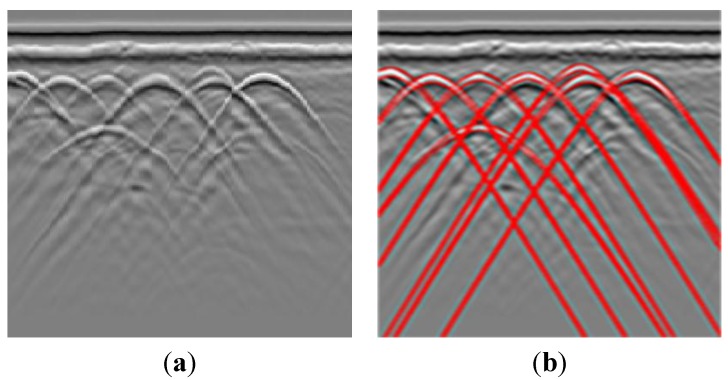
Example of test metal point targets data. (**a**) Real GPR image; (**b**) Processed results with detected patterns.

The last example is shown in Figure 10, which illustrates the effects of the proposed MMSA detector and the final detection results. Note that the proposed detection scheme provides a high detection rate. Figure 10a is a real GPR data profile. It is recorded landmine detection data with a mean frequency of 200 MHz and time window of 100 ns. This is a two dimensional image. The detection result is illustrated in Figure 10b, which shows that all targets are localized.

We compare different aspects of the output of K-means+CEM [12], Hyperbolas Automatic Detection algorithm (HADA) [13] with the MMSA method:

1. How many hyperbolas are recognized correctly (true positives) and how many non-hyperbolas/ non-targets are recognized as hyperbolas (false positives)?
2. The distance error of all correctly recognized targets.

For the first aspect we investigate three different measurements:

(13) $$Recall=\frac{\text{number of true positives}}{\text{number of all actual hyperbolas}}$$

(14) $$Precision=\frac{\text{number of true positives}}{\text{num. of all hyperbolas(true+false positives)}}$$

(15) $$F-measure=2\cdot \frac{Precision\cdot Recall}{Precision+Recal}$$

Table 1 show the different results of MMSA, HADA and K-means+CEM. The comparison in Table 1 shows that MMSA improves Precision and Recall as well as the position estimates. These improvements result from the fact that MMSA detects every hyperbola cluster. For K-means+CEM many position estimates have two errors larger than 10 cm, resulting in bad values for “Recall” and “Precision”. For correctly recognized targets there is still a distance error of a few centimeters. The reason is that K-means+CEM method has problems with nearby and tangent hyperbolas as well as with background noise. For the HADA algorithm, the values for Recall and Precision are higher than with K-means+CEM, but there are still two position estimate errors larger than 10 cm. MMSA achieves much better results than the other methods with the highest values of Recall and Precision.

**Table 1:** Results for data set 1 (*Recall*, *Precision* and *F-measure* with respect to hyperbolas).

Method	Recall (Hyperbolas)	Precision (Hyperbolas)	F-measure (Hyperbolas)	Distance Error(cm)
K-means+CEM	0.62	0.62	0.62	34.8
HADA	0.75	0.75	0.75	22.8
MMSA	1	1	0	5.8

## 5. Conclusions

In this essay, target detection using MMSA has been demonstrated. This method detects buried objects and estimates their position correctly. The method combines the characteristics of wavelet and energy extraction. The amplitude component of the data is extracted using an unsupervised strategy based on MMSA. After the extraction of the amplitude component of the original date, background noise and direct wave are removed to large extent. We use the ROI extraction method to locate the genuine target area among spurious reflections. Then the labeled area is processed by the Hough transform to search the target’s parameters. Finally, we estimate the horizontal and vertical position of the target. Experiments on many different GPR images show that the proposed method provides a general hyperbola-detection system with good results. 
